# Comparing treatment outcomes of different chemotherapy sequences during intensity modulated radiotherapy for advanced N-stage nasopharyngeal carcinoma patients

**DOI:** 10.1186/1748-717X-8-265

**Published:** 2013-11-13

**Authors:** Xueming Sun, Lei Zeng, Chunyan Chen, Ying Huang, Fei Han, Weiwei Xiao, Shuai Liu, Taixiang Lu

**Affiliations:** 1 State Key Laboratory of Oncology in Southern China, Guanggzhou, PR China; 2 Department of Radiation Oncology, Cancer Center of Sun Yat-Sen University, NO.651 Dongfeng east road, Guangzhou City 510060, Guangdong Province, PR China

**Keywords:** Nasopharyngeal carcinoma, Advanced N-stage, Intensity-modulated radiotherapy, Chemotherapy, Prognosis

## Abstract

**Background:**

N-stage is related to distant metastasis of nasopharyngeal carcinoma (NPC) patients. We performed this study to compare the efficacy of different chemotherapy sequences in advanced N-stage (N2 and N3) NPC patients treated with intensity modulated radiotherapy (IMRT).

**Methods:**

From 2001 to 2008, 198 advanced N-stage NPC patients were retrospectively analyzed. Thirty-three patients received IMRT alone. Concurrent chemoradiotherapy (CCRT) was delivered to 72 patients, neoadjuvant chemotherapy (NACT) + CCRT to 82 patients and CCRT + adjuvant chemotherapy (AC) to 11 patients.

**Results:**

The 5-year overall survival rate, recurrence-free survival rate, distant metastasis-free survival rate and progress-free survival rate were 47.7% and 73.1%(p<0.001), 74.5% and 91.3% (p = 0.004), 49.2% and 68.5% (p = 0.018), 37.5% and 63.8% (p<0.001) in IMRT alone and chemoradiotherapy group. Subgroup analyses indicated that there were no significant differences among the survival curves of CCRT, NACT + CCRT and CCRT + AC groups. The survival benefit mainly came from CCRT. However, there was only an improvement attendency in distant metastasis-free survival rate of CCRT group (*p* = 0.107) when compared with RT alone group, and NACT + CCRT could significantly improve distant metastasis-free survival (*p* = 0.017).

**Conclusions:**

For advanced N-stage NPC patients, NACT + CCRT might be a reasonable treatment strategy.

## Background

Although rare among the Caucasian population, nasopharyngeal carcinoma (NPC) is rather common among Asians, especially the Southern Chinese [1]. Intensity-modulated radiotherapy (IMRT) is a major breakthrough in the treatment of NPC. It enables the delivery of higher radiation dose to the lesion while sparing the organs/tissues at risk, thus enhancing the therapeutic ratio, and has been accepted as a more advantageous technique as compared with two-dimensional conventional radiotherapy (2D-CRT). Previous studies have confirmed that IMRT changed the failure pattern of NPC from local recurrence and distant metastasis to predominantly distant metastasis in patients treated with 2D-CRT [2,3].

It is well known that N-stage is related to distant metastasis of NPC patients. Wong et al. [2] reviewed 175 NPC patients treated with IMRT and found that the 3-year distant failure-free survival rate was 95.4%, 100%, 76.6%, and 67.3% for stage N0, N1, N2, and N3 patients, respectively. The Cox proportional hazards regression analysis showed that advanced N-stage was a significant predictor of distant metastasis (p = 0.029). Therefore, it is necessary to explore more effective combined chemoradiation strategies to improve the outcomes of advanced N-stage patients with NPC.

According to the results of previously reported phase III clinical trial and meta-analysis [4-6], concurrent chemoradiotherapy had the most definite survival benefit for locoregionally advanced NPC and had became a standard treatment regimen. However, in these trials and meta-analysis, 2D-CRT technique was adopted for radiotherapy. To our knowledge,there was no phase β clinical trials to identify the value of chemotherapy on locoregionally advanced NPC treated with IMRT. Especially for advanced N-stage patients (whose key problem is distant failure), it is unknown whether chemotherapy could reduce distant metastasis rate and improve treatment outcomes. Therefore, we retrospectively analyzed the long term outcomes of advanced N-stage NPC patients treated with IMRT, and performed the analysis to compare the efficacy of different chemotherapy sequences in these patients.

## Methods

### Patients and patient workup

Between February 2001 and January 2008, 198 histologically proven, newly diagnosed, advanced N-stage NPC patients had undergone full-course definitive IMRT at the Cancer Center of Sun Yat-Sen University of the 198 patients, there were 156 males and 42 females, with a sex ratio of 3.7:1, and the age range was 13 to 78 years (the median age is 41 and there were 3 patients less than 18 years). The routine workup included a complete physical examination, hematologic and biochemistry profiles, fiberoptic endoscope examination of the nasopharynx, magnetic resonance imaging, or contrast-enhanced computed tomography (CT) of the head and neck to accurately evaluate the extent of the primary tumor and regional lymph nodes. Chest radiography, bone scintigraphy, and ultrasonography of the abdominal region were used to exclude distant metastasis. All patients underwent disease staging using the AJCC 2002 staging system. The baseline clinical characteristics are listed in Table 1.

**Table 1:** Baseline characteristics of the 198 patients

**Characteristic**	**IMRT alone**	**CCRT**	**NACT + CCRT**	**CCRT + AC**	**P**
**Age(years)**					0.099
Median	42	44	38	33	
Range	18-78	13-70	16-63	31-57	
**Gender**					0.598
Male	24	56	66	10	
Female	9	16	16	1	
**T stage**					0.097
T1-2	17	28	23	3	
T3-4	16	44	59	8	
**N stage**					0.087
N2	32	62	67	11	
N3	1	10	15	0	
**Histology**					0.740
WHO I	0	0	1	0	
WHO II	5	7	9	0	
WHO III	28	65	72	11	

*Abbreviation*: IMRT alone: intensity-modulated radiotherapy alone; CCRT: concurrent chemoradiotherapy; NACT + CCRT: neoadjuvant chemotherapy and concurrent chemoradiotherapy; CCRT + AC: concurrent chemoradiotherapy and adjuvant chemotherapy.

### Target volume delineation and evaluation of IMRT planning

All patients were immobilized in the supine position with a head, neck, and shoulder thermoplastic mask. CT simulation was performed with a slice thickness of 3 mm extending from the vertex to 2 cm below the clavicle. Both plain CT and contrast-enhanced CT images were obtained and transferred to inverse treatment-planning system (CORVUS 3.0/3.2,Peacock plan) developed by NOMOS Corporation. The primary tumor area and the upper-neck area above the caudal edge of the cricoids cartilage were treated by IMRT. Target volumes were delineated according to our institutional treatment protocol [7], in agreement with the International Commission on Radiation Units and Measurements Reports (ICRU)50 and 62. Planning target volumes (PTVs) for all gross tumor volumes and clinical target volumes (CTVs) were generated automatically after delineation of tumor targets according to the immobilization and localization uncertainties.

Inverse planning was performed on the Corvus System for all patients using Simultaneous Modulated Accelerated Radiation Therapy boost RT [8]. The prescribed dose was 68Gy to the PTV of the GTVnx, 60Gy to the PTV of CTV1, 54Gy to the PTV of CTV2, and 60-66Gy to the PTV of the GTV for the positive cervical lymph nodes in 30 fractions. The prescribed dose to the lower neck and the supraclavicular fossae by irradiation with conventional RT technique was 50Gy/25 fraction for prophylactic intent in whole neck group.

The dose-volume histograms of the treatment targets and critical normal structures were evaluated. For GTV and CTV, the target volumes receiving ≥ 95% of the prescribed dose was used to reflect the target coverage. For the critical organs with functional subunits organized in series, the dose to 5% of the volume couldn’t exceed their tolerance doses. For the critical organs with functional subunits organized in parallel, the dose constraints to 33% of the volume were less than their tolerance doses.

### Chemotherapy

During the study period, we followed our institutional guidelines (Sun Yat-sen University Cancer Center IRB), which recommend both neoadjuvant or adjuvant chemotherapy and/or concomitant chemotherapy for the patients. Deviation from the institutional guidelines were because of organ dysfunction, suggesting intolerance to the chemotherapy, and patient refusal.

Chemotherapy was administered to 165 patients. Concurrent chemotherapy (CCT) was delivered to 72 patients, neoadjuvant chemotherapy (NACT) + CCT to 82 patients and CCT + adjuvant chemotherapy (AC) to 11 patients. The regimens of NACT included TC (paclitaxel 135 mg/m^2^ IV on day 1 and carboplatin AUC = 6 IV on day 1) and PF (cisplatin 80 mg/m^2^ IV on day 1 and 5-Fu 800 mg/m^2^/d continuously IV on day 1–5). There were 33 patients received TC regimen and 49 patients received PF regimen. Regimens were repeated every 3 weeks for 2–3 cycles. CCT included three regimens. Cisplatin (CDDP) 80 mg/m^2^ IV every 3 weeks was delivered to 92 patients, CDDP 30-40 mg/m^2^ IV weekly was delivered to 47 patients, and PF regimen (the same as NACT) was delivered to 26 patients. For patients who received adjuvant chemotherapy, PF regimen (the same as NACT) were repeated every 3 weeks for 2–4 cycles.

### Follow-up and statistical analysis

After RT completion, the patients were subsequently followed up monthly for the first 3 months, every 3 months through 3 years, every 6 months for the next 2 years, and then annually. The median follow-up period was 56 months (range, 3–120 months). The survival time was measure from first day of RT completion to the date of the event or last follow-up visit. The Statistical Package for Social Sciences, version 16.0 (SPSS,Chicago, IL) was used for statistical analysis. The Kaplan-Meier method was used to calculate the overall survival (OS), recurrence-free survival (RFS), distant metastasis-free survival (DMFS), and progress-free survival (PFS) rates. The log-rank test was used to compare the survival curves. All statistical tests were two-sided, and p < 0.05 was considered statistically significant.

## Results

### Treatment outcomes

**Figure 1:**
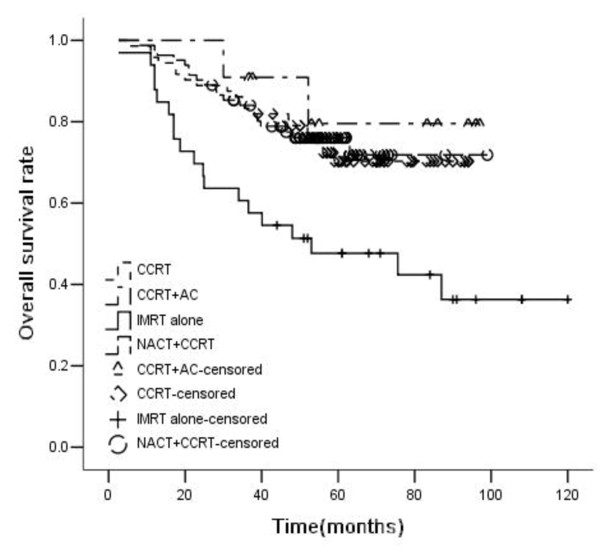
**Overall survival rates of 198 advanced N-stage patients treated with different methods (RT:CCRT*χ***^***2***^ **= 8.942,*P*** **= 0.003; RT:NACT + CCRT*χ***^***2***^ **= 9.357,*P*** **= 0.002; RT:CCRT + AC*χ***^***2***^ **= 4.413,*P*** **= 0.036; CCRT:NACT + CCRT*χ***^***2***^ **= 0.031,*P*** **= 0.860; CCRT:CCRT + AC*χ***^***2***^ **= 0.348,*P*** **= 0.555; NACT + CCRT: CCRT + AC*χ***^***2***^ **= 0.284,*P*** **= 0.594).**

**Figure 2:**
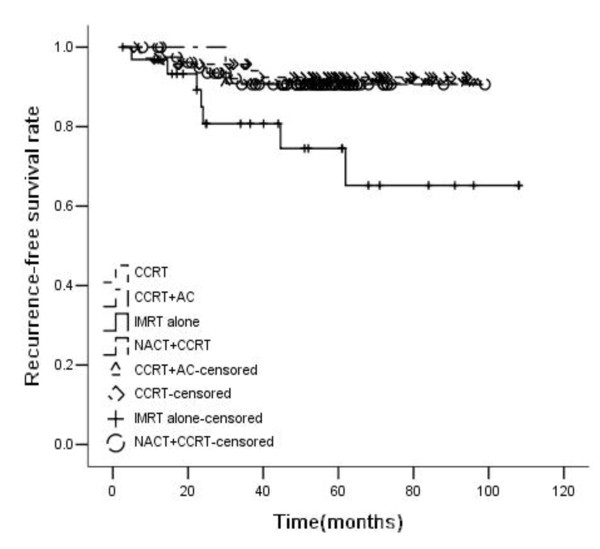
**Recurrence-free survival rates of 198 advanced N-stage patients treated with different methods (RT:CCRT*χ***^***2***^ **= 6.673,*P*** **= 0.010; RT:NACT + CCRT*χ***^***2***^ **= 5.461,*P*** **= 0.019; RT:CCRT + AC*χ***^***2***^ **= 1.621,*P*** **= 0.203; CCRT:NACT + CCRT*χ***^***2***^ **= 0.131,*P*** **= 0.717; CCRT:CCRT + AC*χ***^***2***^ **= 0.062,*P*** **= 0.804; NACT + CCRT: CCRT + AC*χ***^***2***^ **= 0.001,*P*** **= 0.977).**

**Figure 3:**
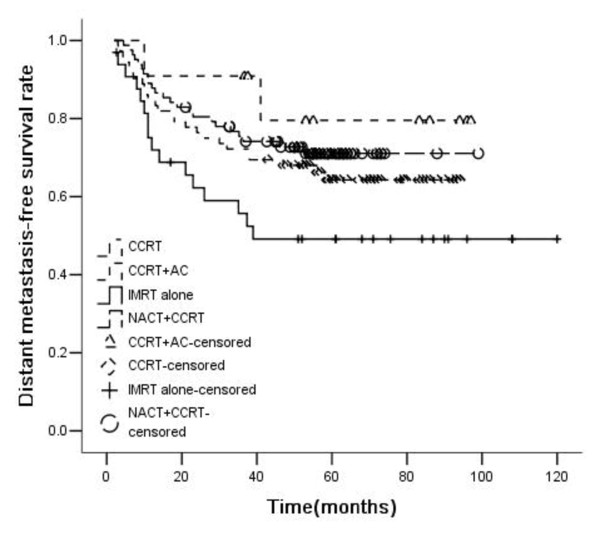
**Distant metastasis-free survival rates of 198 advanced N-stage patients treated with different methods (RT:CCRT*χ***^***2***^ **= 2.600,*P*** **= 0.107; RT:NACT + CCRT*χ***^***2***^ **= 5.694,*P*** **= 0.017; RT:CCRT + AC*χ***^***2***^ **= 3.174,*P*** **= 0.075; CCRT:NACT + CCRT*χ***^***2***^ **= 0.427,*P*** **= 0.672; CCRT:CCRT + AC*χ***^***2***^ **= 0.991,*P*** **= 0.320; NACT + CCRT: CCRT + AC*χ***^***2***^ **= 0.497,*P*** **= 0.481.**

**Figure 4:**
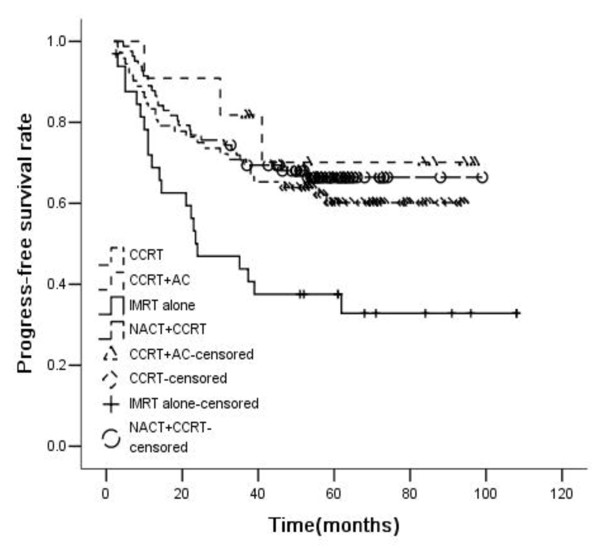
**Progress-free survival rates of 198 advanced N-stage patients treated with different methods (RT:CCRT*χ***^***2***^ **= 6.969,*P*** **= 0.008; RT:NACT + CCRT*χ***^***2***^ **= 10.969,*P*** **= 0.001; RT:CCRT + AC*χ***^***2***^ **= 4.376,*P*** **= 0.036; CCRT:NACT + CCRT*χ***^***2***^ **= 0.481,*P*** **= 0.488; CCRT:CCRT + AC*χ***^***2***^ **= 0.489,*P*** **= 0.484; NACT + CCRT: CCRT + AC*χ***^***2***^ **= 0.182,*P*** **= 0.670).**

For all patients, the 5-year OS, RFS, DMFS, and PFS rates were 69%, 89%, 65%, and 59%, respectively. OS, RFS, DMFS and PFS rates were significantly higher in patients treated with chemoradiotherapy than those treated with IMRT alone (5-year OS rate: 47.7% vs. 73.7%, p<0.001; RFS: 74.5% vs. 91.3%, p = 0.004; DMFS: 49.2% vs. 68.5%, p = 0.018; PFS: 37.5% vs. 63.8%, p<0.001). Patients treated with chemoradiotherapy were divided into three groups: CCRT (Concurrent chemoradiotherapy) group, NACT + CCRT group, and CCRT + AC group. The 5-year OS rates of the three chemoradiotherapy groups were 70.3%, 76.1%, and 79.5%, respectively. Compared with IMRT alone, the OS of these three groups were significantly higher. But there were no statistically significant difference among these three groups. The 5-year RFS rates of the three chemoradiotherapy groups were 92.4%, 90.6%, and 90.9%, respectively. Compared with IMRT alone, CCRT and NACT + CCRT improved RFS significantly. The 5-year DMFS rates of the three chemoradiotherapy groups were 64.3%, 71.1%, 79.5%, respectively. Compared with IMRT alone, only NACT + CCRT could significantly improve DMFS rate, while there was only a trend in CCRT and CCRT + AC group. There was no statistical significance among the difference of the three chemoradiotherapy groups. The 5-year PFS rates of the three chemoradiotherapy groups were 60.2%, 66.4%, and 70.1%, respectively. Compared with IMRT alone, the PFS of these three groups were significantly higher, but there was no statistically significant difference among the three groups (Figures 1, 2, 3 and 4).

At their last follow-up visit, 79 patients had developed treatment failure. Of the 79 patients, 6 had primary recurrence, 3 had regional nodal recurrence, 4 had primary and regional nodal recurrence, and 66 had distant metastasis, respectively.Among them, 3 had developed primary recurrence and distant metastasis, and 4 had developed regional nodal recurrence and distant metastasis. Thirty-five patients had developed distant metastasis in a single organ: 15 cases in bone, 10 cases in liver, 9 cases in lung, and 1 case in mediastinal lymph node. Twenty-four patients had developed multi-organ metastasis. The 79 patients with treatment failure are listed in Table 2.

**Table 2:** Patterns of failure in the 198 patients after treatment

**Patterns of failure**	**IMRT alone**	**CCRT**	**NACT + CCRT**	**CCRT + AC**
**Recurrence**				
Primary recurrence	3	0	3	0
Nodal recurrence	1	2	0	0
Primary, nodal recurrence	1	1	1	1
**Distant metastasis**				
Bone metastasis	3	9	3	0
Lung metastasis	0	2	6	0
Liver metastasis	2	6	3	0
Mediastinal metastasis	1	0	0	0
Mutiple metastasis	8	6	8	2
Distant metastasis, Primary and/or nodal recurrence	2	2	3	0

*Abbreviation*: IMRT alone: intensity-modulated radiotherapy alone; CCRT: concurrent chemoradiotherapy, NACT + CCRT: neoadjuvant chemotherapy and concurrent chemoradiotherapy; CCRT + AC: concurrent chemoradiotherapy and adjuvant chemotherapy.

### Prognostic factors

Univariate analysis by log-rank test revealed that T stage and treatment method were prognostic factors for OS and DMFS. And treatment method was prognostic factor for RFS and PFS (Table 3).

**Table 3:** Univariate analysis for various clinical endpoints

**Characteristic**	**N**	**OS(5-year)**	**p**	**DMFS(5-year)**	**p**	**RFS(5-year)**	**p value**	**PFS(5-year)**	**p**
**Age(years)**			0.448		0.876		0.167		0.743
≤41	100	72.3%		66.8%		85.3%		59.0%	
>41	98	66.2%		64.4%		93.0%		60.4%	
**Gender**			0.451		0.691		0.312		0.665
Male	156	66.5%		65.1%		90.3%		60.7%	
Female	42	78.1%		66.4%		84.7%		54.9%	
**T stage**			0.018		0.048		0.514		0.070
T1-2	71	75.1%		72.1%		93.3%		66.2%	
T3	95	70.9%		67.2%		87.0%		61.0%	
T4	32	48.6%		43.4%		86.5%		39.4%	
**N stage**			0.145		0.125		0.785		0.401
N2	172	71.0%		67.9%		88.9%		61.1%	
N3	26	55.7%		50.1%		91.3%		50.1%	
**Histology**			0.735		0.944		0.821		0.844
WHO I-II	22	71.3%		67.9%		88.9%		63.3%	
WHO III	176	68.6%		65.2%		89.1%		59.1%	
**Treatment method**			<0.001		0.018		0.004		<0.001
IMRT alone	33	47.7%		49.2%		74.5%		37.5%	
Chemoradoitherapy	165	73.1%		68.5%		91.3%		63.8%	

All characteristics of survival were analyzed by COX regression model. Adjusted by age, gender and histology, multivariate analysis showed that T stage, N stage and treatment method were independent prognostic predictors of OS, DMFS and PFS. Only treatment method was independent prognostic predictors of RFS (Table 4).

**Table 4:** Multivariate analysis for various clinical endpoints

**Endpoint**	**Characteristic**	**HR**	**95.0% CI**	**P**	
RFS	Treatment method	0.667	0.467	0.953	0.026
DMFS					
	Treatment method	0.694	0.561	0.859	0.001
	N stage	2.560	1.304	5.029	0.006
	T stage	1.772	1.246	2.522	0.001
PFS					
	Treatment method	0.674	0.556	0.818	<0.001
	T stage	1.645	1.190	2.275	0.003
	N stage	2.004	1.039	3.867	0.038
OS	Treatment method	0.621	0.497	0.775	<0.001
	N stage	2.782	1.372	5.642	0.005
	T stage	1.902	1.321	2.740	0.001

*Abbreviation*: HR: hazard ratio; CI: Confidence interval.

### Toxicity

Treatment-related toxicity was scored according to the Radiation Therapy Oncology Group radiation morbidity scoring criteria. For the patients treated with IMRT alone, the most frequently observed acute toxicity was mainly grade 1 or 2 xerostomia and mucositis. The incidence of acute grade 3 mucositis was 24.2%. Six patients (18.2%) had grade 1 leukopenia and/or neutropenia. No grade 4 acute toxicities were observed. For the patients treated with chemoradiotherapy, seventy-six patients (46.1%) had acute grade 3 mucositis and 34 patients (20.6%) had grade 3 vomiting. Seventeen patients developed grade 3 and 3 patients developed grade 4 neutropenia. And 9 patients had grade 3 or 4 thrombocytopenia. The most common severe acute hematologic toxicities (grade ≥ 3) were leukopenia and/or neutropenia n = 20 (30.8%) and thrombocytopenia n = 9 (5.5%).

Late toxicities were assessed in 152 patients (26 patients in the IMRT alone group and 126 patients in the chemoradiotherapy group) with ≥ 2 years follow up. The most common late toxicities were grade 1 or 2 xerostomia, tympanitis, and subcutaneous fibrosis. No patient had grade 4 late toxicity (Table 5).

**Table 5:** The Frequency of late toxicities for the patients after treatment

**Toxicity**	**IMRT alone (n = 26)**			**Chemoraditherapy (n = 126)**		
	**Grade 1 no.(%)**	**Grade 2 no.(%)**	**Grade 3 no.(%)**	**Grade 1 no.(%)**	**Grade 2 no.(%)**	**Grade 3 no.(%)**
Xerostomia	14(53.8)	1(3.8)	0(0)	64(50.8)	36(28.6)	0(0)
Tympanitis	9(34.6)	7(26.9)	0(0)	50(39.7)	38(30.2)	2(1.6)
Subcutaneous fibrosis	10(38.5)	7(26.9)	0(0)	54(42.8)	39(31.0)	2(1.6)
Trismus	2(7.7)	0(0)	0(0)	7(5.6)	0(0)	0(0)
Temporal lobe injury	1(3.8)	1(3.8)	0(0)	7(5.5)	2(1.6)	0(0)

## Discussion

Radiotherapy is the principal treatment modality for non-metastasis NPC. N-stage is a prognostic factor of distant metastasis of NPC patients, no matter which radiation technique we used, the advanced N-stage patients had a high distant metastasis rate [2,9-12] (Table 6).In the present study, of 198 advanced N-stage NPC patients treated with IMRT, stage N2 and N3 patients with 5-year OS, RFS, DMFS, PFS of 71.0%, 88.9%, 67.9%, 61.1%, and 55.%, 91.3%, 50.1%, 50.1%, respectively. Although IMRT achieved an excellent locoregional control, the DMFS and OS are still low, and similar to the patients undergoing 2D-CRT in the past studies [9-11]. Lai et al. [11] compared the results of IMRT with those of 2D-CRT in the treatment of NPC patients and found that IMRT did not improve distant control of all N category tumors, the reason might be the nasopharyngeal carcinoma have a higher probability of micrometastatic dissemination at the time of initial diagnosis, and until effective methods to treat disseminated disease are developed, the effect of local control on survival would not be readily discerned. The high distant metastasis rate may be one reason of the low OS rate. Moreover, in the present study, OS, RFS, DMFS and PFS rates were significantly higher in patients treated with chemoradiotherapy than those treated with IMRT alone. Although to our knowledge,there was no randomized phase III study to confirm the value of chemotherapy on locoregionally advanced NPC treated with IMRT, according to our results, chemotherapy still play an important role in the treatment of advanced N-stage NPC patents.

**Table 6:** Summary of the Studies Reporting DMFS and OS of advanced N-stage NPC patients

**Author**	**Year**	**N**	**Technique of RT**	**DMFS(%)**	**OS(%)**
Leung, et al [9]	2005	1070	2D-CRT	5-year:	5-year:
				N_2_:71.6	N_2_:60.3
				N_3a_:66.9	N_3a_:48.4
				N_3b_:52.1	N_3b_:40.2
Ma, et al [10]	2001	621	2D-CRT	5-year:	—
				N_2_:62	
				N_3_:51	
Lai, et al [11]	2011	1276	2D-CRT	5-year:	—
			IMRT	2D-	
				CRT:N_2_:75.1	
				N_3_:65.4	
				IMRT: N_2_:80.5	
				N_3_:60.9	
Wong, et al [2]	2010	175	IMRT	3-year:	—
				N_2_:76.6	
				N_3_:67.3	
Yi, et al [12]	2008	147	IMRT	3-year:	3-year:
				N_2-3_:65.2	N_2-3_:68.5
Current study	2012	198	IMRT	5-year:	5-year:
				N_2_:68.5	N_2_:71.0
				N_3_:50.1	N_3_:55.7

Intergroup study 0099 [4] demonstrated that CCRT + AC is superior to radiotherapy alone for locoregionally advanced NPC patients with respect to PFS and OS. However, the phase III randomized study in endemic regions did not achieve similar results [13]. Recently, a comparison between concurrent chemoradiotherapy followed by adjuvant chemotherapy and concurrent chemoradiotherapy was performed in 508 locally advanced NPC patients enrolled in a phase III trial conducted by Chen et al. [14]. In this trial, concurrent chemoradiotherapy followed by adjuvant chemotherapy did not significantly improve PFS. The meta-analysis by Baujat et al. [5] indicated that the OS benefit is 6% with the use of chemotherapy, and the benefit is largely owing to the CCRT. No evidence of prognostic benefit was observed with adjuvant chemotherapy. The other meta-analysis [6] also demonstrated that CCRT was the most effective treatment modality for the improvement of survival. However, most patients in the former studies were treated with 2D-CRT. But IMRT could increase the dose conformity and irradiate higher dose to the treatment targets, so the locoregional control was improved significantly [15,16]. Therefore, the margin of benefit potentially gained with additional chemotherapy maybe reduced. Lin et al. [17] have questioned the role of concurrent chemotherapy in advanced NPC patients treated with IMRT. In the present study, compared with IMRT alone, CCRT could significantly improve OS, RFS, PFS of advanced N-stage patients. The possible reason is most advanced N-stage patients also with advanced T-stage. Hara et al. [18] found that although treated with IMRT, the local failure rate was still more than 10% in stage T3/4 NPC patients. Larger tumors are related to adverse radiobiological parameters, including an increased clonogen number [19,20] and hypoxia[21,22], which are difficult to overcome with dose escalation alone. In this case, concurrent chemotherapy could still enhance the radiosensitivity to improve locoregional control. And we also observed that compared with IMRT alone, there was only a trend toward a lower distant failure rate with CCRT, but the correlation did not reach statistical significance (p = 0.107). CCRT alone is ineffective in reducing distant metastasis maybe is a reasonable explanation [23]. Moreover, we found even though CCRT + AC group have higher OS, RFS and PFS than IMRT alone group, the differences of survival rates between CCRT + AC and CCRT group did not reach statistical significance. So maybe the survival benefit of CCRT + AC is largely owing to the CCRT. Adjuvant chemotherapy did not improve prognosis yet. But although the survival rates of CCRT + AC group did not reach statistical significance when compared with CCRT or NACT + CCRT group, we observed this group had the highest survival rates in the three groups. This phenomenon maybe explained by the small number of patients treated with CCRT + AC, and the same N2 stage of all the 11 patients.

The role of neoadjuvant chemotherapy followed by concurrent chemoradiotherapy or RT is a matter of outstanding interest. The pool data analysis by Chua et al. [24] found NACT + RT arm was associated with a significant improvement in locoregional relapse-free survival and disease-specific survival in advanced-stage NPC, but not in OS. Hui et al. [25] published the results of a randomized phase II trial in which stage III-IVb NPC patients were randomly assigned to receive either NACT + CCRT, or CCRT alone. A positive impact on survival was observed, since the 3-year overall survival for the NACT + CCRT versus the CCRT arm was 94.1% vs.67.7% (p = 0.012).The present study demonstrated that the differences of survival rates between NACT + CCRT and CCRT groups did not reach statistical significance. So the survival benefit of CCRT + AC maybe is mainly owing to the CCRT. However, compared with IMRT alone, NACT + CCRT could improve DMFS rate significantly, while there was only a trend with CCRT. Maybe in the condition of such a high locoregional control by CCRT, NACT + CCRT is unlikely to improve RFS rate significantly. But because of more powerful than concurrent chemoradiotherapy, NACT might be effective in controlling micrometastatic dissemination.

## Conclusion

In this study, we have described the long-term outcomes for patients with advanced N-stage NPC who underwent IMRT with the SMART boost technique. Compared with IMRT alone, CCRT could significantly improve OS, RFS, PFS of advanced N-stage patients, and NACT + CCRT could significantly improve DMFS. There was no evidence of survival benefit was observed with adjuvant chemotherapy in this study. So, according to the results of the present study, NACT + CCRT might be a reasonable strategy for advanced N-stage NPC patients. However, our study was limited by the small sample size, use of retrospective analysis, and possible selection bias during the matching process. As a result, relative randomized clinical trial is still needed in the future. In addition, although an excellent locoregional control was achieved by IMRT, the DMFS and OS were not improved compared with the advanced N-stage patients undergoing 2D-CRT. New strategies combining different treatment modalities to reduce the rate of distant metastasis effectively also need to be developed in the future.

## Competing interests

The authors declare that they have no competing interests.

## Authors’ contributions

TL designed the study. XS, LZ, CC, YH, FH, WX, and SL collected the data. TL, XS, LZ and CC did the data analysis, interpretation, and wrote the report. TL, XS, and LZ did the statistical analysis. All authors read and approved the final manuscript.
